# Mapping as a knowledge translation tool for Ontario Early Years Centres: views from data analysts and managers

**DOI:** 10.1186/1748-5908-3-4

**Published:** 2008-01-18

**Authors:** Anita Kothari, S Michelle Driedger, Julia Bickford, Jason Morrison, Michael Sawada, Ian D Graham, Eric Crighton

**Affiliations:** 1 Faculty of Health Sciences, University of Western Ontario, Arthur & Sonia Labatt Health Sciences Building, Room 222, N6A 5B9, London, Ontario, Canada; 2 Department of Community Health Sciences, University of Manitoba, S113-750 Bannatyne Ave, R3E 0W3, Winnipeg, Manitoba, Canada; 3 Department of Biosystems Engineering, University of Manitoba, E2-376 Engineering Building, University of Manitoba, R3T 5V6, Winnipeg, Manitoba, Canada; 4 Laboratory for Applied Geomatics and GIS Science (LAGGISS), Department of Geography, University of Ottawa, K1N 6N5, Ottawa, Ontario, Canada; 5 School of Nursing and Dept of Epidemiology & Community Medicine, University of Ottawa – 451 Smyth Road, K1H 8M5, Affiliate Scientist, Clinical Epidemiology Program, Ottawa Health Research Institute, VP Knowledge Translation, Canadian Institutes of Health Research, Ottawa, Ontario, Canada; 6 Department of Geography, University of Ottawa, K1N 6N5, Ottawa, Ontario, Canada

## Abstract

**Background:**

Local Ontario Early Years Centres (OEYCs) collect timely and relevant local data, but knowledge translation is needed for the data to be useful. Maps represent an ideal tool to interpret local data. While geographic information system (GIS) technology is available, it is less clear what users require from this technology for evidence-informed program planning. We highlight initial challenges and opportunities encountered in implementing a mapping innovation (software and managerial decision-support) as a knowledge translation strategy.

**Methods:**

Using focus groups, individual interviews and interactive software development events, we taped and transcribed verbatim our interactions with nine OEYCs in Ontario, Canada. Research participants were composed of data analysts and their managers. Deductive analysis of the data was based on the Ottawa Model of Research Use, focusing on the innovation (the mapping tool and maps), the potential adopters, and the environment.

**Results:**

Challenges associated with the innovation included preconceived perceptions of a steep learning curve with GIS software. Challenges related to the potential adopters included conflicting ideas about tool integration into the organization and difficulty with map interpretation. Lack of funds, lack of availability of accurate data, and unrealistic reporting requirements represent environmental challenges.

**Conclusion:**

Despite the clear need for mapping software and maps, there remain several challenges to their effective implementation. Some can be modified, while other challenges might require attention at the systemic level. Future research is needed to identify barriers and facilitators related to using mapping software and maps for decision-making by other users, and to subsequently develop mapping best practices guidelines to assist community-based agencies in circumventing some challenges, and support information equity across a region.

## Introduction

Community-based child health agencies may be rich in timely, context-specific data, while at the same time face significant hurdles in translating their raw data into meaningful evidence for informed decision-making. The use of maps and mapping is increasingly recognized as a key knowledge translation tool to assist in the transformation of local data for decision-making [1]. The World Health Organization states that "mapping of events not only facilitates epidemiological analysis but is also effective for advocacy, informing the pubic and generating action by decision-makers" [2]. Mapping offers a visual representation of data. Instead of searching through pages of tables and graphs, copious amounts of information can be understood quickly in a single map. Like a metaphor, a map allows viewers to easily grasp the relationships between distinct sets of information.

Community agencies are beginning to recognize the value of maps [3], and there are a growing number of contexts and configurations in which community agencies can obtain access to geographic information systems and mapping [4]. While the technology may be available, it is less clear what users – those producing maps and those making decisions using maps – require from this technology for evidence-informed program planning and policy development. This paper presents findings from the first phase of a two-phase qualitative study with ten Ontario Early Years Centres' (OEYCs) child data analysts and eight OEYC managers in Southwestern Ontario, Canada. OEYCs, which are funded by the government, provide free programs and activities for children aged infant to six years and their parents/caregivers, to contribute to Ontario children's healthy early development. As such, these programs aim to positively impact lifelong learning and health [5-7]. OEYCs first opened in 2002 and now include 103 main sites in addition to several smaller satellite sites across Ontario. The research question that this project phase sought to answer was: What are the barriers and facilitators related to using mapping software and maps for decision-making in OEYCs? This project involved a web-based customized mapping program developed in consultation with users. In this paper, we report on some of the challenges regarding GIS and mapping as expressed by participants in interviews, focus groups, and prototype software demonstration workshops.

## Methods

Phase I of this project is based on the theoretical underpinnings of ethnography, in which we explored the cultural context of the Data Analyst Coordinators (DACs) [8-10]. In particular, we were interested in exploring how the culture of OEYCs influenced the perceptions, beliefs, and attitudes toward the mapping program. The Ottawa Model of Research Use (OMRU) guided data collection and analysis [11,12]. This model focuses on three important factors that are integral to research or innovation uptake: the innovation itself, the potential adopter, and the environment in which the innovation is being introduced. Perceptions of the attributes or characteristics of the mapping software and maps can influence potential adopters' decisions to use these tools in either positive or negative ways. Potential adopters (the data analysts and managers) have particular motivations, skills, and attitudes that may affect uptake. As well, the environment contains structural, organizational, and social influences that may foster or impede the uptake of the innovation.

### Sample

A purposive sampling strategy was employed in this project. In particular, the research team recruited some OEYCs who had earlier sought assistance for mapping through the former Central West Health Planning Information Network. Working with these OEYCs allowed this project to build on an existing, collaborative, organizational-level relationship (none of the individual participants in our current sample were involved earlier). These organizations have been introduced to the idea of mapping tools, and consequently potentially represent the critical sub-population Rogers [13] calls "early adopters" of innovations – those most likely to take up an innovation and be able to provide early impressions about possible barriers and facilitators. Other OEYCs in Southern Ontario were also invited to participate. Due to computer-related server limitations, we needed to ensure that we could support the invited OEYCs. Ten OEYC data analysts and eight managers participated (representing eight teams; two of the eight teams have two data analysts and one manager, and the other six teams have one data analyst and one manager). Initially, twelve OEYC data analyst/manager dyads were asked to participate and four declined. The reasons given for declining included vacancies in the data analyst position, and already having access to a commercial GIS tool.

### Data collection

Phase I data collection took place between November 2004 and October 2006 and involved focus groups, telephone interviews, and feedback during 'hands-on' development workshops. In this way, there were multiple opportunities for participants to engage, in a step-wise fashion, in the design of the mapping tool. We started with focus groups to attain a preliminary understanding of the needs and preferences of the participants. Then we had a hands-on workshop to allow them to engage with a prototype mapping tool. Following this, we conducted telephone interviews in order to identify further comments based on the workshop experience. The development of the mapping software program EYeMap, designed specifically for this study, followed a participatory design process [14]. EYeMap is a web-based mapping tool enabling a secure interface to facilitate the uploading of proprietary data. It was developed using the University of Minnesota's MapServer [15,16]; Open Source Geospatial Foundation, 2006; and map tool resources. Mapserver is fully Open Geospatial Consortium (OCG) compliant and as such, easily interacts with other standard web mapping services and software. In particular, resources at MapTools.org, hosted by DM Solutions Group were heavily leveraged in the development cycle. We used MapServer in a Linux environment in combination with Perl and C++ to build the prototype mapping of EYeMap for the OEYC data analysts (see [17]). As a web-based solution, all thematic data (*e.g*., census boundaries, socioeconomic data, etc.) are maintained on the server side. In effect, this frees users from data formatting, processing, maintenance, and other issues. As such, the producers and users of maps in OEYCs were fully engaged in the design process so that the final product was tailored to their specific needs and concerns.

The participatory design process for data analysts began with an initial meeting with data analysts and managers in 2004. The purpose of this initial meeting was to further describe the research project, to develop strong relationships and trust among the participants, to identify key areas of concern for data analysts regarding mapping and existing mapping technologies, to address any other concerns of the analysts, and to collect a partial 'wish list' of what a web-based mapping tool would comprise. Data analysts also participated in two additional half-day workshops with the development team to further refine the possibilities of a web-based solution against the desired 'wish list'.

Once a proof of concept was fully developed, we held our first set of focus groups, one with OEYC data analysts (n = 9) and one with managers (n = 8). As per the OMRU, these focus groups served to identify the perceptions and attitudes toward maps and mapping; the motivations, skills, and attitudes of the DACs and managers; organizational supports or limitations; and other environmental factors that could facilitate or impede the use of mapping.

To further capture individual experiences and perspectives of the managers, individual telephone interviews were conducted in the weeks following this initial focus group. These interviews were approximately 20 minutes in length and asked managers to comment on: the types of decision-making they are involved in, the types of information they use to inform decisions, how information is conveyed and presented, any prior experiences with mapping (challenges or limitations), and the nature of communication between managers and data analysts.

Finally, data analysts were involved in two separate day-long training sessions for each release phase of EYeMap. Following the first training day, analysts were able to test out the software prototype back at their home agency and provide feedback and suggestions for further changes that would suit their needs. Participants were encouraged to frequently contact the project staff to describe any difficulties or successes they were experiencing with mapping. Project staff also initiated email contact with participants to provide updates on the software development and to invite participants to share comments or questions. These changes were systematically logged. Ethics approval for this research was obtained from the University of Ottawa (ethics #: 05-04-16).

### Data analysis

The combination of several data collection methods (focus groups, individual interviews, interactive design meetings) enabled data to be triangulated for confirmability [18,19]. In qualitative research, threats to validity are two fold: threats to description and interpretation [20]. To maintain accurate description, all data were digitally recorded and transcribed verbatim. Participants were asked for clarification when any questions arose around the meaning of text segments in the transcripts in order to make sure that we were interpreting their comments reasonably. As well, the principal investigators took field notes during these sessions to capture the suggestions and comments of participants. A common coding template was developed based on the OMRU [21,22], using QSR Nvivo 2 to support deductive analysis of the data. All of the transcripts were coded by one of the authors (JB) following the constructs of the OMRU framework. The codes were then read by another team member (AK, MD) to ensure that there was consistency across transcripts. The emerging patterns were discussed, challenged and interpreted (JB, AK, MD) at a peer debriefing session.

## Results

### The innovation

#### Perceptions and beliefs about other mapping software

Potential users of an innovation frequently have previously established perceptions of the innovation. While they may not have direct personal experience with a particular innovation, they may have heard about it through word of mouth, or have used a similar innovation that serves as a mental proxy.

Several participants described the limitations of other mapping software they had used, in particular one mapping program to which several data analysts had access. One data analyst described the inability of this program to present multiple layers of data in a single map. As a result, she had to use multiple maps to convey information that she felt should be conveyed in a single map. She explained:

'It has its limitations in layering data and just visually as well if you want to show multiple sets. So sometimes you need multiple maps instead of having to layer them overtop. That's the biggest thing.'

Others described available mapping programs as 'too basic'. Other reported limitations included the difficulty involved in placing a title on a map: Data analysts continuously need to re-type the title 'over and over again' as the software did not save the title; there was no print-preview option; and, the font was difficult to change. Data analysts also described the difficulty in zooming in and out. These functional limitations associated with other mapping software may influence the users' decision to adopt the EYeMap tool.

#### Prohibitive cost

Several of the participants described the cost of market-based mapping software as prohibitive. In addition to buying the software, participants also noted the high cost required to train data analysts to use the software.

'...[F]or our own OEYCs to try to access any mapping is a real challenge and it's a cost issue on top of that, a time issue.'

'MapInfo is available. But training on the software is very difficult and that is expensive. One person in the organization has the skills so we have to go to her. No manual available. Just the training and the software would be wonderful.'

As a result of not being able to afford in-house map-making, OEYCs are forced to contract out their map-making. Consequently, participants experienced long lag times between ordering and receiving maps:

'Just maybe in the fact that since she is not doing the maps herself, there might be a little bit of lag time because of course you are competing with other departments who also want maps made. So there would be the time constraints that we would probably be put a little lower in the queue compared to planners or real-estate people, farmers, that kind of thing.'

Some participants could not afford market-based GISs and the training these programs require. The lack of accessibility of these other GIS packages due to financial constraints, and the undesirability of contracting out map-making, may support positive perceptions towards the introduction of the (free) EYeMap tool.

#### Perceptions related to the EyeMap prototype

In this project, the innovation is the web-based mapping software and maps, and the OMRU suggests that user expectations will make uptake of the tool more or less likely. In this case, participants anticipated that the maps would influence and justify decisions. For example, one manager who did not yet have mapping capabilities in her organization explained:

'I would rely on maps insofar as if we have to justify the types of programs in a community...or if I'm looking at new programs I am going to say 'According to the current mapping that we have of people using our facilities, there is a big gap over there.'

Another participant wanted to adopt the tool because she believed it would improve efficiency in the workplace:

'...As far as being able to have a ... more effective look at what is happening with our community, and if there are friends in our various neighborhoods where we either need more services, less services, we need to look at providing service in more effective way, or reallocate services. Our hope is that at the end of the day, this work will assist us in doing work more efficiently and more effectively.'

It became apparent in the focus group with managers that participants had different notions of how mapping could best be integrated into OEYCs. The differences appeared to pivot around the variations in opinion regarding how and where mapping knowledge should be situated within the organization. For example, one participant felt strongly that the mapping software would need to be adequately 'user-friendly' so that all employees within the organization would be able to access and use spatial data and then produce maps. The participant explained:

'I thought that's what this was for, was to build capacity with a small little agency to be able to upload their own participation data and create a map all by themselves, with no involvement of the DAC [data analyst], no involvement from the planning department, no one having to pay two hundred dollars for a map.'

At a fundamental level, this participant believed that knowledge gained from mapping should build community capacity and be accessible by all. She continued:

'It's the old proverb, give a person a fish or teach them how to fish. And that's what we are trying to do. We are trying to build this evidence-based planning capacity in communities by not just training the DAC [data analyst], but training all service providers.'

Other participants disagreed and felt that mapping was under the responsibility of data analysts and should remain exclusively within their portfolio. Thus, the tools will be introduced to potential users who hold strong views about how the mapping software and maps should be best used within their organization. If the tools are not designed in a way that reflects those preconceived ideas about the innovation, then the potential adopters may choose not to implement them. In this study, the mapping tool was tailored for data analysts to use. As a result, one participant decided not to adopt the tools because they were not introduced in a way that met her expectations. Namely, the software was designed to be used by someone with some quantitative expertise, and not for everybody within the OEYC organization.

This project incorporated a participatory design process in an attempt to effectively respond to the needs and expectations of users such that they would be more likely to adopt the final tool. This novel process – closely intertwined with the development of the innovation – invoked some comments. Participants stated that they appreciated having their comments and feedback taken into consideration throughout the development of the tool. They felt that this process resulted in a prototype that was more tailored to their requirements. However, participants also commented that the drawback with this participatory design process is that it takes more time for the final product to be ready for implementation and use.

### Potential adopters

Potential adopters in this project included the map producers and those who use maps to make decisions (managers). Each has particular motivations, skills, and attitudes that may affect uptake of the innovation. Previous positive experiences with mapping surfaced in the data. General attitudes about the steep learning curve related to mapping software, lack of skills to accurately interpret maps, and confusion about the data analyst's role were challenges faced by potential adopters in this study.

#### General attitudes about mapping software

Some participants were already actively producing and interpreting maps themselves, while others were eager to begin. One manager stated, 'I understand the value of a mapping format.' Data analysts were described as 'chomping at the bit to start using the mapping tool'.

Despite volunteering to be involved in this project, some participants also perceived mapping as a time-consuming and difficult skill to learn. For example, one manager described the experiences of her previous data analyst in trying to learn how to use a (different) mapping software program in the past. She explained:

'It's my belief that the Ministry did provide some training with software which our very first data analyst coordinator took...she was totally frustrated by the process...Then I hired the DAC [data analyst] that I have now and it was, by that time, it didn't seem pertinent and it didn't seem necessary and we didn't have time to deal with it regardless.'

This participant went on to explain, 'Based on that amount of work, the whole mapping thing really went off our radar. We did not use it.' Thus, although participants were positive about the new innovation, based on past experiences, some participants felt that the training required to master the software required a certain time commitment.

#### Lack of skills to accurately interpret the maps

Another frequently discussed challenge attributed to potential adoptors was acquiring the skills and experience necessary for accurate interpretation of maps. Managers reflected on the importance of having extensive knowledge about the community represented in a map. Many managers had experienced the puzzlement of looking at a map that did not reflect the perceived reality of that community:

'And we looked at this map of the city of X, and it appeared that there were a large number of children who were living in poverty in the south end of X, which really didn't make a lot of sense to us. Anybody who has been to X, knows it has a huge build up of large homes and people that commute to [a large urban centre] for work. So we started looking at that and thinking, "Well, this just can't be. So what does it mean?" And the only thing we could figure, and this is just our thinking, not based on anything that any expert has told us, is that the census tract does include pockets at the top of the geographical area of some low income housing and co-op apartments and that sort of thing. But if you were to just look at that map, because where it plotted the big circle of children living in poverty, it just did not initially make any sense at all. I would see that as a bit of a limitation. You'd really need to know your community to figure out a puzzle like that.'

They also noted the difference in interpretation of the same data by two different analysts:

'...And of course what they, the joint advisory committee, want to see are stats. So we are providing stats and we've got two DACs [data analysts] that are providing those stats. And again, [DAC 1] is interpreting it one way, [DAC 2] is interpreting the other way, and it has taken... it's been two and a half years and we are still trying to get the definitions because ... it's all interpretation.'

Thus, having the expertise to appropriately interpret maps and related data was a concern for participants, and may play a role in the subsequent uptake of the innovation.

#### Confusion over DAC role

Another challenge pertaining to the potential adopter was the inconsistency and variation in the conceptualization and definition of the role of data analysts. According to some participants, data analysts each have specific job descriptions tailored to their local community needs. As a result some data analysts are less trained than others to engage in mapping:

'And again, the other thing, the DACs [data analysts] were hired and there wasn't a similar job description for the DACs either. So some of them have very different skills, you know? And as you meet the DACs, you realize that they have different focuses and different skills and that's amazing to see. That's fine too. They suit their community. But it's amazing that when I went looking for a DAC, I had my vision of what I wanted whereas somebody else had their vision.'

The managers described a confusion regarding what data analysts do:

'Because rollout of DACs was different, different ways of operating. No one checks on them so no one ever knows what they are doing. No one has brought them together.'

As a result, some managers find it difficult to compare their programs or services with those of other OEYCs:

'And I think if the definition and the understanding isn't the same across the board, then for me to compare what we are doing at X, to what you're doing in Y, is not going to be an accurate comparison because there is different interpretations as far as what you are charting.'

This possible inconsistency raises concerns in terms of the quality of data 'products', like maps, that can be offered to each community for planning and coordination efforts.

### Environment

A number of potential barriers were identified regarding the context in which the researchers attempted to implement the innovation. Some of these issues are beyond the researchers' control, although they may impact on the willingness to adopt the innovation. The challenges related to the environment included: collecting accurate data, confidentiality issues associated with small rural populations, and usefulness of data requested for reporting and evaluation.

A major environmental challenge that participants discussed was that of collecting and interpreting data. This involved the difficulty in accessing accurate data, confidentiality issues for rural areas, and the risk of misrepresentation or inaccurate interpretation of maps.

The issue of accessing accurate data was a key point in many of the interviews and focus groups. Some of the participants described systemic problems, such as having to rely on outdated 2001 Census data that no longer captured the reality of their communities. One participant explained:

'So we find that very difficult...because the data is so old. We had 16,000 births last year in the region of X. And we have one community that, in 2001, shows that there is no population zero-to-six in one area. And yet we know that there is a huge population; [it's] because the census is so old.'

Other participants described issues that were particularly pertinent to OEYCs located in rural communities. For example, a reliance on postal code data was problematic:

'We were developing some maps through GIS for Best Start to support the analysis that we'd done, and our DAC had then put all the data necessary via postal code. And when the map came out, we looked at it and went, "Well, it makes no sense!" because we know the community. And of course, in a rural area, postal code...'

The only type of geographic data available is often at the level of the postal code, and in rural areas this covers a large area and is not specific enough to be useful. This represents a larger systemic problem of the environment into which the new innovation was being introduced.

#### Confidentiality issues

Another issue, which was identified by participants located in rural areas, was that of confidentiality and small numbers of clients. One participant stated:

'The other limitation is our small numbers. So sometimes mapping doesn't make sense when you can simply show your stats when we're talking eight kids... So that is also just a confidentiality issue. We have to remember that.'

In this situation, maps may be a less effective and less appropriate knowledge translation tool. Thus, a rural setting could influence whether or not a mapping tool was adopted.

#### Usefulness of data requested for reporting and evaluation

Another environmental challenge was the disconnect between The Ontario Ministry of Children and Youth Services reporting requirements and the reality of working at the local level in OEYCs. For example, several participants questioned the usefulness and meaning of data that they were required to report. One manager felt the routine indicators that she reported were not meaningful, and in some cases sufficiently difficult to quantify, that she reported random numbers rather than accurate data:

'They don't really tell a good story. I mean, to be honest... I am sure everyone else did, pick the number out of the air when they first started, and now they give them quarterly. And sometimes they are totally different from what you started with, but it really doesn't mean anything.'

Another manager concurred that:

'It's just absolutely ridiculous. How many times can you count water or water play in a center? I just stick 50 down and go with that the rest of the year. I don't bother because it's ridiculous.'

This challenge is an aspect of the larger environmental context influencing the implementation of the innovation. The OEYC managers would prefer to report on information that is meaningful, and many of the examples they identified as meaningful would require the use of mapping tools. For example, managers described the geographic catchment area from which a particular program is drawing clients as being important information. This challenge may facilitate the successful adoption of the innovation.

## Discussion

The purpose of this paper is to identify the key challenges and facilitators for OEYC data analysts and managers in the uptake of mapping and mapping software. Data collection included a variety of qualitative methods including individual interviews, focus groups, and interactive 'hands-on' software design workshops. The findings are interpreted using the OMRU framework. As such, the challenges are grouped into three domains that consist of the innovation (mapping software and maps), the potential adopters (data analysts and managers), and the environment.

Several of the challenges that emerged from the data are modifiable and therefore represent facilitators for EYeMap uptake. For example, the development of EYeMap, a web-based customized mapping program based on a participatory design process with associated interventions (*e.g*., provision of training), mitigates the time and cost challenges associated with a steep learning curve and software training related to commercially available GIS; we make this claim based on our ongoing successful partnership with users. The challenges related to the ability to interpret maps and data might also be addressed with spatial literacy interventions that accompany GIS software. Other studies have illustrated similar modifiable challenges in community-based GIS research projects [1]. In particular, Buckeridge *et al*. [23] described how they dealt with issues such as how to facilitate appropriate interpretation of data and maps, and identifying and acquiring data. The next phase of this project will attempt to work with these modifiable challenges in order to support the adoption of this knowledge translation intervention.

Other challenges that emerged from the data are not modifiable with any GIS tool. For example, access to accurate and recent census data, inconsistencies in the definition of the data analyst role, and usefulness of data required for provincial reporting are systemic difficulties embedded within a larger socio-political landscape that cannot be resolved through a GIS tool, regardless of attempts to tailor its features.

It is important to understand and identify these modifiable and non-modifiable challenges because they might result in important variations among OEYCs in their ability to analyze, display and manipulate spatial data. Among our participants, some OEYCs did not have any mapping capabilities, while others have the ability to use the most sophisticated market-based GIS programs (*e.g*., ArcGIS). GIS and mapping are knowledge translation tools that can facilitate optimal program and policy decisions to positively impact local communities. Given that OEYCs influence the way public services for children are offered, there might be significant implications for the gap between the information haves and have-nots. A decade ago, Sawicki and Craig [24] voiced their concern for the democratization of data, stating, 'community groups from low-income neighborhoods have the most to gain from full access to data, yet the least capability to achieve that access or make use of the data once they have it.' Similarly, Harris and Weiner [25] described their concern that GIS technology 'has the potential to disenfranchise the weak and not so powerful through the selective participation of groups and individuals'.

A unique and important approach in this study is the involvement of decision-makers (managers) in the intervention design process. Like Scotch [26], we see the issue as one of problem-solving for planning and evaluating community-based services. That is, the issue is how to translate local data into knowledge for decision-making. Similar to more formal spatial decision support systems, the implementation of EYeMap will be complemented by a tailored support system for managers. McLafferty [1] describes the two directions that spatial data decision-making projects are moving towards: the first direction incorporates an array of technological tools (*e.g*., algorithms, optimal location modeling), while the second direction focuses on the human aspects of knowledge translation (*e.g*., participatory approaches, data dissemination, local concerns). This study focuses on the human dimension of decision-making, where maps are a potentially useful tool for this purpose.

Another strength related to the findings of this study is that they are theoretically informed by the OMRU. As a qualitative study, the findings are specific to the sample and context described herein. They do, however, provide hypotheses for other researchers to pursue in alternate settings. As a limitation, one might argue that the OEYCs in the sample who were considered experienced or early adopters of GIS may have identified different barriers and challenges than would a sample of participants from less supportive OEYCs. This may be less of an issue at second glance – only three OEYCs were 'experienced', and furthermore, at the individual level, none of the participants were involved in the earlier project. In fact, as evident by the variation in findings, the participants reflected a range of experiences. Thus, this study provides a starting point for other health-related mapping projects.

## Conclusion

Several challenges associated with GIS and mapping were identified by a sample of managers and data analysts in Ontario Early Years Centres. The challenges were identified using the OMRU framework and pertained to the innovation, the potential adopter, and the environment. Some of these challenges can be modified through the use of easily accessible mapping software accompanied by support interventions, while others require attention at a larger systemic level and cannot be solved with a mapping tool.

## Competing interests

The author(s) declare that they have no competing interests.

## Authors' contributions

AK and MD were involved in the conceptualization, data collection, interpretation and writing portions of this manuscript. JB was involved in data collection, analysis, and writing portions of the manuscript. JM and MS were involved in the software development, conceptualization and writing portions of the manuscript. IG was involved in the conceptualization of this project, and provided the "senior voice" throughout. EC was involved in the interpretation of data and manuscript preparation. All authors read and approved the final manuscript.

## References

[B1] McLafferty S (2003). GIS and Health Care. Annual Review of Public Health.

[B2] Public Health Mapping and GIS. http://www.who.int/health_mapping/tools/en/.

[B3] Elwood S (2006). Beyond cooptation or resistance: urban spatialpolitics, community organizations, and GIS-based spatial narratives. Annals of the Association of American Geographers.

[B4] Leitner H, Elwood S, Sheppard E, McMaster S, McMaster R (2000). Modes of GIS provision and their appropriateness for neighborhood organizations: examples from Minneapolis and St. Paul, Minnesota. URISA Journal.

[B5] Frank J, Mustard F (1994). The determinants of health from a historical perspective. Daedalus.

[B6] Mustard F Early childhood development andexperience-based brain development: the scientific underpinnings ofthe importance of early childhood development in a globalized world.

[B7] Mustard F, McCain MN, Bertrand J (2000). Changing beliefs tochange policy: the early years study. ISUMA.

[B8] Creswell J (1998). Qualitative inquiry and research design: choosing among five traditions.

[B9] Creswell J (1998). Research design: Qualitative, quantitative, and mixed methods approaches.

[B10] Tedlock B, Denzin N, Lincoln Y (2000). Ethnography and ethnographic representation. Handbook of Qualitative Research.

[B11] Graham I, Logan J (2004). Innovations in knowledge transfer and continuity of care. Canadian Journal of Nursing Research.

[B12] Logan J, Graham ID (1998). Toward a comprehensive interdisciplinary model of health care research use. Science Communication.

[B13] Rogers EM (1995). Diffusion of innovations.

[B14] Bloomberg J, Giacomi J, Mosher A, Swenton-Wall P, Schuler D, Namioka A (1993). Ethnographic field methods and their relation to design. Participatory Design: Principles and Practices.

[B15] Kropla B (2005). Beginning MapServer: open source GIS development.

[B16] Boulos M, Honda K (2006). Web GIS in practice: publishing our health maps and connecting to remote Wms sources using open source UMN Mapserver and DM Solutions Maplab. International Journal of Health Geography.

[B17] Driedger S, Kothari A, Morrison J, Sawada M, Crighton E, Graham I (2007). Using participatory design to develop public) health decision support systems through GIS. International Journal of Health Geographics.

[B18] Denzin N, Lincoln Y (1998). The Landscape of Qualitative Research: Theories and Issues.

[B19] Marshall C, Rossman G (2006). Designing Qualitative Research.

[B20] Patton M (2002). Qualitative Research and Evaluation Methods.

[B21] Logan J, Graham ID (1998). Toward a comprehensive interdisciplinary model of health care research use. Science Communication.

[B22] Graham I, Logan J (2004). Innovations in knowledge transfer and continuity of care. CJNR.

[B23] Buckeridge DL, Mason R, Roberson A, Frank J, Glasier R, Purdon L, Amrhein CG, Chaudhuri N, Fuller-Thomson E, Gozdyra P, Hulchanski D, Moldofsky B, Thompson M, Wright R (2002). Making health data maps: a case study of a community/university research collaboration. Social Science & Medicine.

[B24] Sawicki DS, Craig WJ (1996). The democratization of data: bridging the gap for community groups. Journal of the American Planning Association.

[B25] Harris T, Weiner D (1998). Empowerment, marginalization, and "community-integrated" GIS. Cartography & Geographic Information Systems.

[B26] Scotch M, Parmanto B, Gadd CS, Sharma RK (2006). Exploring the role of GIS during community health assessment problem solving: experiences of public health professionals. International Journal of Health Geographics.

